# BlendMol: advanced macromolecular visualization in Blender

**DOI:** 10.1093/bioinformatics/bty968

**Published:** 2018-11-27

**Authors:** Jacob D Durrant

**Affiliations:** Department of Biological Sciences, University of Pittsburgh, Pittsburgh, PA, USA

## Abstract

**Summary:**

Programs such as VMD and PyMOL are excellent tools for analyzing macromolecular structures, but they do not implement many of the advanced rendering techniques common in the film and video-game industries. In contrast, the open-source program Blender is a general-purpose tool for industry-standard rendering/visualization, but its user interface is poorly suited for rigorous scientific analysis. We present BlendMol, a Blender plugin that imports VMD or PyMOL scenes into Blender. BlendMol-generated images are well suited for use in manuscripts, outreach programs, websites and classes.

**Availability and implementation:**

BlendMol is available free of charge from http://durrantlab.com/blendmol/. It is written in Python.

**Supplementary information:**

Supplementary data are available at *Bioinformatics* online.

## 1 Introduction

Molecular modeling plays a prominent role in biological and chemical research. While mathematical analysis is critical, it remains true that many insights come only from visual inspection. Programs dedicated to macromolecular visualization and analysis (e.g. VMD and PyMOL) (DeLano, 2002; Humphrey *et al.*, 1996) generate images that are scientifically rigorous and esthetically pleasing. Artistic considerations are far from frivolous. Poor esthetics are distracting and so impede scientific communication. Advanced rendering techniques such as photo-realistic shadows and lighting can convey subtle but important structural information.

**Fig. 1.**
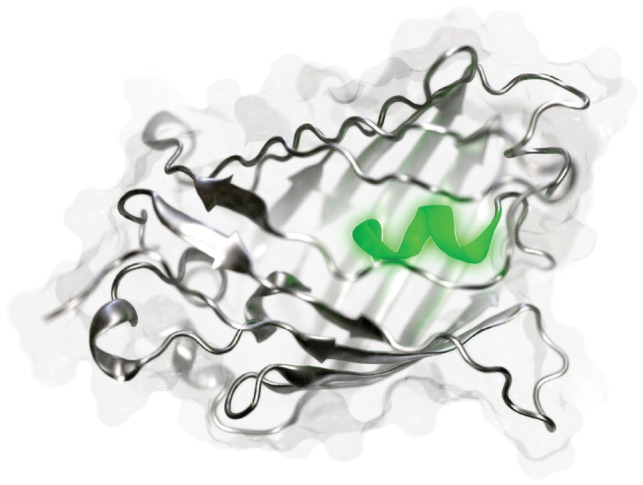
A green fluorescent protein with a highlighted central helix (Color version of this figure is available at *Bioinformatics* online.)

Beyond macromolecular visualization, the film and video-game industries have also done much to advance computer graphics in recent years. The free, open-source program Blender implements many industry-standard rendering tools. Though popular molecular-visualization programs have impressive rendering engines (Stone *et al.*, 2013), they cannot yet match Blender (Fig. 1).

General-purpose modeling programs such as Blender are thus increasingly popular tools for producing photo-realistic macromolecular images and videos (Johnson and Hertig, 2014). These programs allow users to position macromolecules in their actual microscopic contexts, providing much needed scientific perspective (see, e.g. the June 2, 2017 and May 6, 2016 covers of *Science*) (Hastie *et al.*, 2017; Stepanek and Pigino, 2016). Companies such as Digizyme and Random42 produce and sell these visualizations. And universities ranging from Harvard Medical School to the Cleveland Institute of Art teach classes in macromolecular visualization.

Several Blender plugins can load molecular models independent of any dedicated molecular-visualization program (Andrei *et al.*, 2012). Given that VMD and PyMOL have been in development for roughly 20 years, independently developed Blender plugins typically lack many of their powerful visualization features. Efforts to implement these features demand a complex, difficult-to-maintain codebase, perhaps explaining why so many previously published molecular-visualization plugins are no longer functional. Beyond that, the Blender UI itself, while well suited for visualization, is not convenient for macromolecular analysis. Researchers will likely analyze their molecular systems in programs such as VMD or PyMOL regardless.

Our past macromolecular-visualization workflow has thus required us to switch between VMD and Blender. We first analyze our molecular structures in VMD. Then, when preparing a final image, we separately reconstruct the VMD-visualized scene in Blender. This reconstruction process is tedious. Both VMD and PyMOL can export molecular meshes to Blender-compatible file formats. But after export, these files must be separately imported into Blender. Both VMD and PyMOL save meshes using camera coordinates rather than the world coordinates of the models themselves, so two imported molecular meshes will not align unless the same camera position is used on export. Overcoming this challenge requires TCL or Python scripts that standardize the camera position. Finally, the exported meshes themselves are often suboptimal. For example, we routinely have to remove duplicate mesh vertices within Blender after import. A better approach is to create a plugin that seamlessly bridges VMD/PyMOL and Blender. Importing VMD/PyMOL scenes into Blender should take minimal effort, and steps such as adjusting coordinates and optimizing mesh geometries should be automated.

We here present BlendMol, a Blender plugin that interfaces with the VMD or PyMOL executable to easily import VMD Visualization State and PyMOL Session files. One can also work entirely within Blender, without ever opening a dedicated macromolecular-visualization program. If the user provides a Protein Data Bank (PDB) ID or a PDB file, the plugin uses VMD or PyMOL to automatically generate a simple, default visualization.

The BlendMol plugin has been tested in Blender 2.79 on Windows 10 Home (1709), macOS High Sierra (10.13.3) and Ubuntu Linux (16.04 LTS). We release it under the terms of the GNU GPL Version 3 license. A copy is available free of charge from http://durrantlab.com/blendmol/. The Supplementary Material includes detailed installation instructions (Supplementary Video S1), as well as examples of use.

## 2 Software comparison

To understand its utility, it is helpful to compare BlendMol to other programs for macromolecular visualization.

### 2.1 Desktop programs

BlendMol and dedicated programs such as VMD (Humphrey *et al.*, 1996) or PyMOL (DeLano, 2002) do not occupy the same niche. The later include tools for analyzing atomic-resolution structures and simulations. In contrast, BlendMol serves as a bridge between VMD/PyMOL and Blender. Within Blender, molecules are stored as meshes, not collections of atoms with exact coordinates. Rather than analysis, the goal is to create enhanced images for use in journals, outreach programs, websites and classes.

### 2.2 Browser-based molecular-visualization libraries

The Supplementary Material shows how BlendMol can facilitate browser-based macromolecular visualization. In that sense, it is similar to JavaScript libraries such as 3Dmol.js (Rego and Koes, 2015), NGL Viewer (Rose and Hildebrand, 2015; Rose *et al.*, 2016) and JSmol/Jmol (Hanson, 2010; Hanson *et al.*, 2013). These libraries are ideal for rapidly generating browser-based molecular representations on the fly. They process input (PDB) files directly, without requiring intermediate programs such as VMD or Blender.

BlendMol is useful when visualizations can be pre-rendered. For example, Blender can ‘bake’ photorealistic shadows/lighting onto browser-compatible image textures (Supplementary Fig. S4). In contrast, JavaScript-only libraries cannot perform these resource-intensive calculations in real time. Furthermore, to the best of our knowledge, no browser-based macromolecular-visualization library can display molecules in virtual reality (Supplementary Fig. S5). BlendMol, together with the Babylon.JS game engine (see Supplementary Material), provides a more immersive experience.

### 2.3 Other modeling-software plugins

To our knowledge, no other plugin so seamlessly integrates popular macromolecular-analysis and computer-graphics programs (e.g. VMD/PyMOL and Blender). BlendMol allows users to import into Blender the very representations and coloring schemes they use for analysis in VMD or PyMOL. Advanced rendering thus becomes but another step in their existing workflows. That having been said, there are several other molecular-visualization plugins for Blender and similar 3D modeling environments. The Supplementary Material includes a detailed comparison.

## 3 Conclusion

BlendMol bridges popular macromolecular-analysis programs and the open-source, general-purpose modeling program Blender. It allows scientists to visualize molecules using state-of-the-art techniques, thus furthering collaborations and inspiring students. The Supplementary Material describes general limitations and future directions. It also shows how advanced lighting/materials can improve realism and communicate scientific information (Supplementary Fig. S2). BlendMol also facilitates 3D printing (Supplementary Fig. S3), in-browser visualization (Supplementary Fig. S4), virtual reality (Supplementary Fig. S5), video rendering (Supplementary Fig. S6 and Video S2) and molecular-dynamics visualization (Rajendiran and Durrant, 2018) (Supplementary Videos S3 and S4).

## Supplementary Material

bty968_Supplementary_Data

## References

[bty968-B1] Andrei R.M. et al (2012) Intuitive representation of surface properties of biomolecules using BioBlender. BMC Bioinf., 13, 1471–2105. 10.1186/1471-2105-13-S4-S16 PMC3434447 22536962

[bty968-B2] DeLano W.L. (2002) The PyMOL molecular graphics system. http://pymol.org (10 December 2018, date last accessed).

[bty968-B3] Hanson R.M. (2010) Jmol–a paradigm shift in crystallographic visualization. J. Appl. Crystallogr., 43, 1250–1260.

[bty968-B4] Hanson R.M. et al (2013) JSmol and the next‐generation web‐based representation of 3D molecular structure as applied to proteopedia. Isr. J. Chem., 53, 207–216.

[bty968-B5] Hastie K.M. et al (2017) Structural basis for antibody-mediated neutralization of Lassa virus. Science, 356, 923–928. 28572385 10.1126/science.aam7260 PMC6007842

[bty968-B6] Humphrey W. et al (1996) VMD: visual molecular dynamics. J. Mol. Graphics, 14, 33–38. 10.1016/0263-7855(96)00018-5 8744570

[bty968-B7] Johnson G.T., Hertig S. (2014) A guide to the visual analysis and communication of biomolecular structural data. Nat. Rev. Mol. Cell Biol., 15, 690–698. 25245078 10.1038/nrm3874

[bty968-B8] Rajendiran N., Durrant J.D. (2018) Pyrite: a blender plugin for visualizing molecular dynamics simulations using industry-standard rendering techniques. J. Comput. Chem., 39, 748–755. 29280166 10.1002/jcc.25155

[bty968-B9] Rego N., Koes D. (2015) 3Dmol.js: molecular visualization with WebGL. Bioinformatics, 31, 1322–1324. 25505090 10.1093/bioinformatics/btu829 PMC4393526

[bty968-B10] Rose A.S. et al (2016) Web-based molecular graphics for large complexes. In: *Web3D ‘16 Proceedings of the 21st International Conference on Web3D Technology, July 22–24, 2016,* ACM, Anaheim, California, pp. 185–186.

[bty968-B11] Rose A.S., Hildebrand P.W. (2015) NGL Viewer: a web application for molecular visualization. Nucleic Acids Res., 43, W576–W579. 25925569 10.1093/nar/gkv402 PMC4489237

[bty968-B12] Stepanek L., Pigino G. (2016) Microtubule doublets are double-track railways for intraflagellar transport trains. Science, 352, 721–724. 27151870 10.1126/science.aaf4594

[bty968-B13] Stone J.E. et al (2013) GPU-accelerated molecular visualization on petascale supercomputing platforms. In: *8th International Workshop on Ultrascale Visualization, November 17 2013*, ACM, Denver, CO, US, p. 6.

