# Supplementary material for: BlendMol: advanced macromolecular visualization in Blender
Source: Bioinformatics. 2018 Nov 27;35(13):2323–5. doi: 10.1093/bioinformatics/bty968 (PMC6596883; doi:10.1093/bioinformatics/bty968)
Supplement: bty968_Supplementary_Data [file bty968_supplementary_data.zip › bty968-Suppl_data/Supplementary_Data.docx]

| Applications Note  BlendMol: Advanced Macromolecular Visualization in Blender  Jacob D. Durrant^1,*^  ^1^Department of Biological Sciences, University of Pittsburgh, Pittsburgh, Pennsylvania 15260, United States.  *To whom correspondence should be addressed. |
| --- |


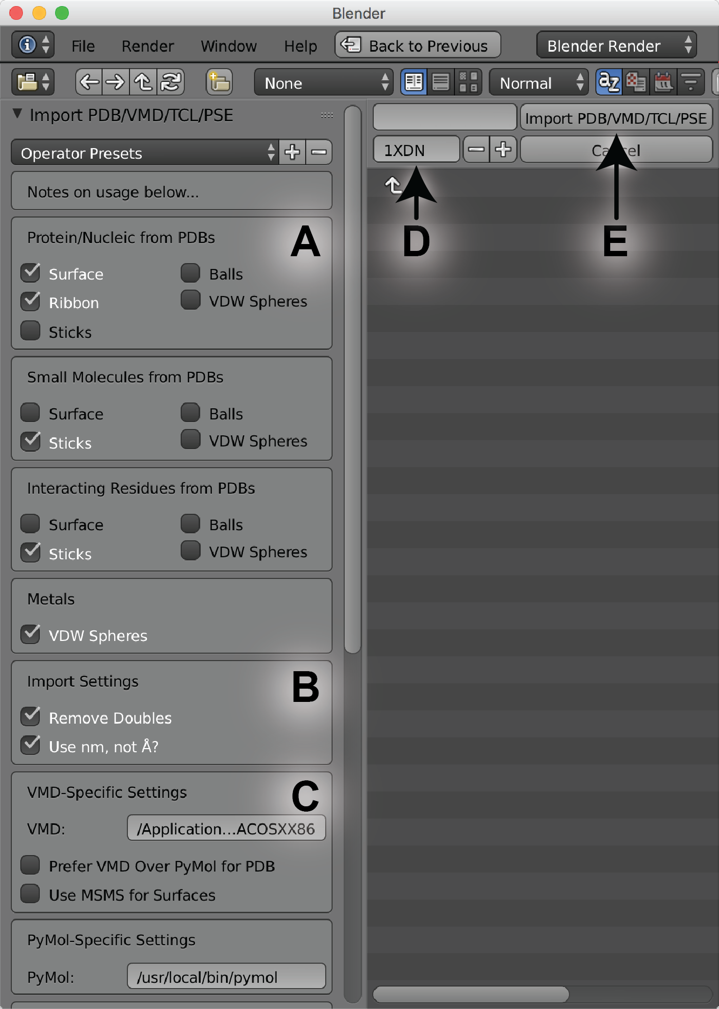
**Fig. S1. The BlendMol import dialogue.** A) Settings to control PDB representations. B) Additional options to process imported meshes. C) Settings to control the VMD and PyMOL executables. D) The “File path” text box. E) The Import Button.

# Installation

To install the BlendMol plugin, first download a copy of the ZIP file from http://durrantlab.com/blendmol/. Within Blender, click the “File > User Preferences….” menu item to open the “Blender User Preferences” dialogue. Then access the add-ons panel by clicking the “Add-ons” button. Specify the location of the ZIP file using the “Install Add-on from File….” button. Activate the plugin by clicking the “Import-Export: BlendMol - PDB/VMD/PyMOL” checkbox. To automatically activate every time Blender starts, click the “Save User Settings” button.

Critical plugin preferences can be set from the Add-ons panel by clicking the expanding carat. To use VMD and PyMOL, respectively, the user must specify the absolute paths to each executable. If only one program is required, the path to the other can be left unchanged. VMD state files (.vmd/.tcl) and PyMOL session files (.pse) are imported via their respective programs. But both programs can visualize local and remote PDB files. Checking “Prefer VMD Over PyMOL for PDB” will force the plugin to load PDB files using VMD. If the user has installed the MSMS surface representation (Sanner, et al., 1995) within VMD, she may also wish to check “Use MSMS for Surfaces.” Video S1 illustrates plugin installation and usage.

# Import Dialogue: Options

To use BlendMol, open the import dialogue by clicking the “File > Import > PDB/VMD/PyMOL (.pdb, .vmd, .tcl, .pse)” menu item. This dialogue allows the user to set additional plugin preferences and to import PDB, VMD state, and PyMOL session files.

The left panel includes additional user preferences (Fig. S1). Many control how the plugin instructs VMD or PyMOL to create models from PDB files, which do not include the molecular-representation details that are present in VMD state and PyMOL session files. Available options allow the user to control the modeling of macromolecules (proteins and nucleic acids), small molecules, macromolecule residues that directly interact with small molecules, and metal atoms. These components can be rendered as surfaces, ribbons, sticks, balls, and van der Waals (VDW) spheres, as appropriate (Fig. S1A). We provide these PDB options for convenience. Users who require greater control over the generated meshes should import state and session files created in VMD and PyMOL, respectively.

Two additional options allow the user to control mesh import, regardless of the external molecular-visualization program used. If “Remove Doubles” is checked, BlendMol will automatically merge duplicate vertices present in imported meshes. This feature adds to the import time, but the resulting meshes are more memory efficient and less prone to artifacts when manipulated within Blender. The user can also control the scaling of the imported meshes. By default, BlendMol uses Ångstroms as the unit of measurement to maximize compatibility with the PDB file format. If “Use nm, not Å?” is checked, nanometers are used instead (Fig. S1B).

Finally, the import-dialogue panel also includes the options present in the User Preferences dialogue described above (e.g., full paths to VMD/PyMOL executables, etc., Fig. S1C).

# Import Dialogue: Importing

After setting the plugin options, the user indicates what to import using the “File path” text box near the top of the dialogue (Fig. S1D). If the user enters a PDB ID, BlendMol will automatically download the appropriate PDB file form the Protein Data Bank (Berman, et al., 2000). Otherwise, the user should indicate the location of a local PDB file (.pdb), VMD state file (.vmd or .tcl), or PyMOL session file (.pse). Pressing the “Import PDB/VMD/TCL/PSE” button begins the import process (Fig. S1E). BlendMol automatically runs VMD/PyMOL in the background. It 1) normalizes the VMD/PyMOL camera position, 2) renders molecular meshes to Blender-compatible files, 3) imports those files into Blender, and 4) optionally optimizes mesh geometries.

# Vertex-Based Atomic-Property Visualization

VMD can color macromolecular surfaces according to properties such as crystallographic B-factors, partial atomic charges, element identities, and electrostatic potentials. These coloring schemes are also accessible within Blender via BlendMol. To illustrate, we first loaded a crystal structure of influenza neuraminidase (PDB ID: 2HU4:A) (Russell, et al., 2006) into VMD and shaded the surface by the B-factors (white to black). We then imported the saved VMD state file into Blender using BlendMol.

To demonstrate Blender-specific rendering features, we converted the B-factor colors to luminosities. In the Blender Internal Render engine, we first transferred (i.e., “baked”) material colors to vertex colors and then cleared the VMD-derived material slots. We next switched to Blender’s physics-based Cycles renderer and used the vertex colors to mix Diffuse BSDF (bidirectional scattering distribution function) and Emission shaders via a Mix Shader. Surface luminosity thus varies according to the underlying B-factors, from high luminosity (small B-factors) to low luminosity (large B-factors, Fig. S2). Any two Cycles materials can be similarly mixed (e.g., glossy and diffuse materials, glass and metallic materials, etc.). Adobe Photoshop’s color-adjusting and filtering tools were used to finalize the Blender-rendered image.


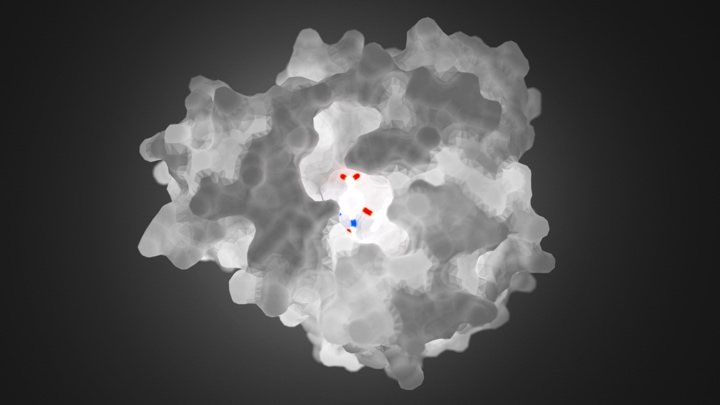


**Fig. S2. Influenza neuraminidase.** Surface luminosity rendered according to the crystallographic B-factors.

# Illustrating Protein Function via Enhanced Lighting

Standard macromolecular-visualization programs allow some control over lighting and shadows. But Blender implements many of the advanced lighting algorithms that professional computer-graphics artists use. To illustrate, we imported a VMD-generated model of a green fluorescent protein (PDB ID: 4XOW) (Duwe, et al., 2015) into Blender. Fig. 1 shows how Blender's enhanced rendering features can facilitate scientific communication. The central helix that holds the chromophore in place is rendered in a glowing green material.

This image also illustrates other rendering techniques available in Blender’s Cycles Renderer. The metallic ribbon representation uses Blender’s Principled BSDF shader, which draws on computer-graphics research done at Disney (Doppioslash, 2018; McAuley, et al., 2012). The protein surface again shows how two Blender shaders can be mixed. When combined, the Glass BSDF and Transparent BSDF shaders produce a translucent surface that does not obscure the underlying ribbon. Blender’s Mist Pass and Camera Depth of Field are also useful for highlighting certain image regions. The Mist Pass was here used to fade the image to white, per the camera-vertex distances. Depth of Field subtly blurs image regions distant from the central helix, mimicking tilt-shift photography. Photoshop was again used to finalize the image.

# 3D Printing


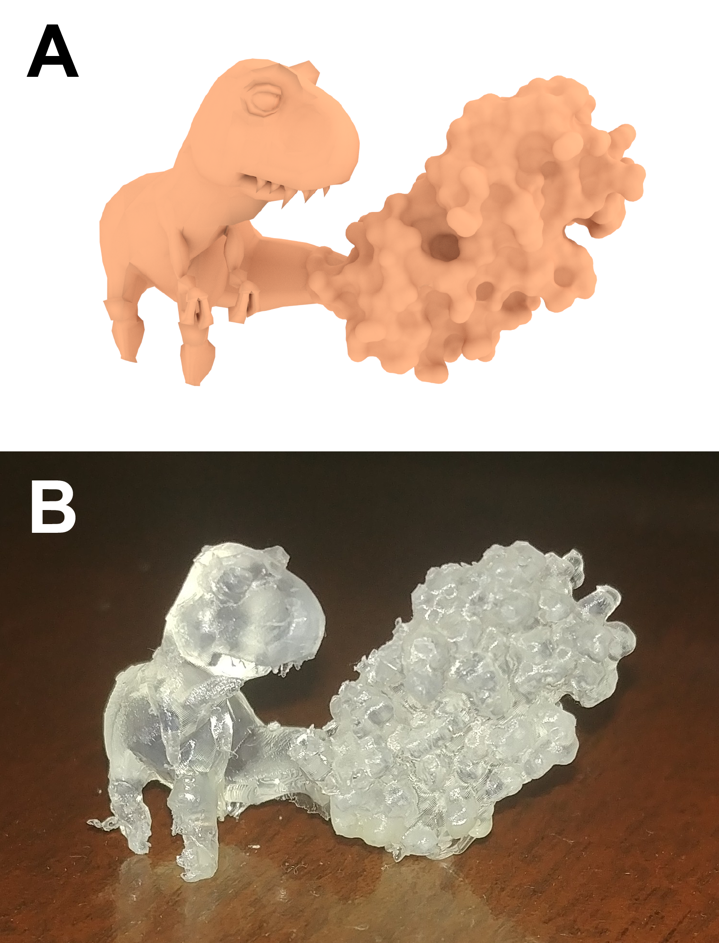


**Fig. S3.** **Once imported into Blender, macromolecular meshes can be artistically modified before 3D printing.** The public-domain dinosaur was obtained from Blend Swap. BlendMol generated the mesh of LARP1. A) A rendered image. B) A rough-draft 3D-printed model.

BlendMol also facilitates 3D printing. Blender’s “3D Print Toolbox” add-on can prepare BlendMol-imported macromolecular meshes for printing. There are, of course, other methods for printing 3D molecules (Scalfani, et al., 2016). But Blender allows for artistic modification of the mesh.

To illustrate, we obtained a public-domain dinosaur model from Blend Swap, a community-driven online repository. We then imported a mesh of the LARP1 DM15 domain (PDBID: 5C0V) (Lahr, et al., 2015), a potential anti-cancer drug target in the mTORC1 pathway. In this case, we provided BlendMol with only a PDB ID, skipping direct VMD visualization entirely. The plugin automatically interfaced with VMD via a command shell to create the models. After posing the dinosaur appropriately, we fused the LARP1 model to its tail using Blender’s Boolean Modifier. Blender’s 3D Print Toolbox, together with PreForm, a stand-alone program by Formlabs, further prepared the model for 3D printing (Fig. S3). The model was printed at Open Lab, part of the University of Pittsburgh’s Center for Teaching and Learning.

# Browser-Based Visualization

BlendMol also enables advanced browser-based macromolecular visualization. Photorealistic lighting and shadows require calculations that are generally too intense for a browser. But Blender can save (or “bake”) these elements onto a simple image. When mapped onto the meshes, the images reproduce the pre-calculated visuals without requiring the same intense calculations. Painting this pre-rendered image onto a protein mesh within a browser is entirely feasible.

To illustrate, we used BlendMol to import a mesh of the PsbO subunit of Photosystem II (PDB ID: 5G38) (Bommer, et al., 2016). We selected this protein because of its interesting beta-barrel fold. In this example, BlendMol interfaced with PyMOL to generate the macromolecular meshes. We manually UV unwrapped the meshes in Blender, such that each mesh vertex mapped to a unique 2D image coordinate.

The Principled BSDF shader applied metallic materials to the protein surface and ribbon meshes. To enhance surface textures, we also used bump mapping to modify the normal vectors used to calculate lighting. The resulting render appears to have complex surface details that are not present in the actual mesh. With these details in place, we baked the metallic materials, lighting, and shadows of each mesh onto separate image textures.


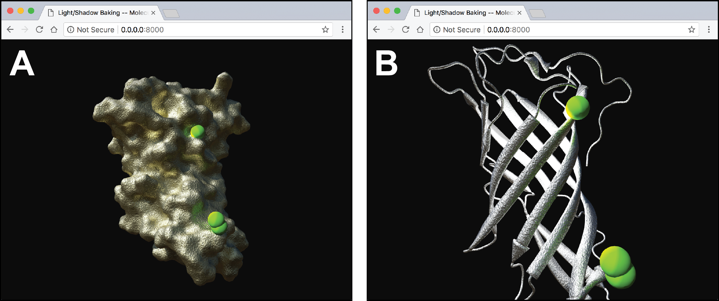


**Fig. S4.** **Advanced 3D macromolecular visualization in the browser.** Photorealistic lighting and shadows are pre-calculated in Blender to improve browser-based performance. A) At a distance, the protein is rendered in the surface representation. B) When the user zooms, the ribbon representation fades in.

We used Babylon.JS, an open-source, WebGL-based 3D engine, to visualize the protein meshes in a browser. Babylon.JS provides a Blender exporter that easily converts Blender scenes to the web-compatible babylon format. Using Babylon.JS, JavaScript, HTML5, and CSS, we created a simple web app that displays the scene in a browser (Fig. S4, see Code S5 for the source code). To illustrate the advantages of interactive browser-based visualizations, the surface representation fades to ribbon as the user zooms in. A live demo can be found at http://durrantlab.com/apps/blendmol/web/baked-lighting-shadows/

It is important to note that BlendMol is not a web-first technology. BlendMol models must first be exported to a web-appropriate format (e.g., the babylon format). The user must then create her own HTML5/JavaScript/CSS web page to deliver those models to site visitors via the browser. Code S5 should provide a helpful starting point.

# Virtual Reality

BlendMol also enables virtual-reality (VR) macromolecular visualization. In recent years, commercial VR headsets have brought this technology to the mainstream. Some companies have even produced cheap, mobile-phone-compatible headsets made of cardboard. VR visualization can serve powerful collaborative and educational purposes. Babylon.js also brings VR to the web browser. One key advantage of browser-based VR is that users need not download and install separate programs. If they have a VR headset, viewing the 3D content is as simple as visiting a website.

To illustrate, we downloaded a public-domain model of the Galleria dell'Accademia di Firenze from Blend Swap. We replaced Michelangelo's David with the ribonuclease inhibitor-angiogenin complex (PDB ID: 1A4Y) (Papageorgiou, et al., 1997), selected for its interesting structure. To optimize online delivery, we used Blender’s “Remove Doubles” and “Decimate Modifier” tools to simplify the geometry. We also replaced the works of classical art displayed on the Galleria walls with computer-rendered images of proteins.


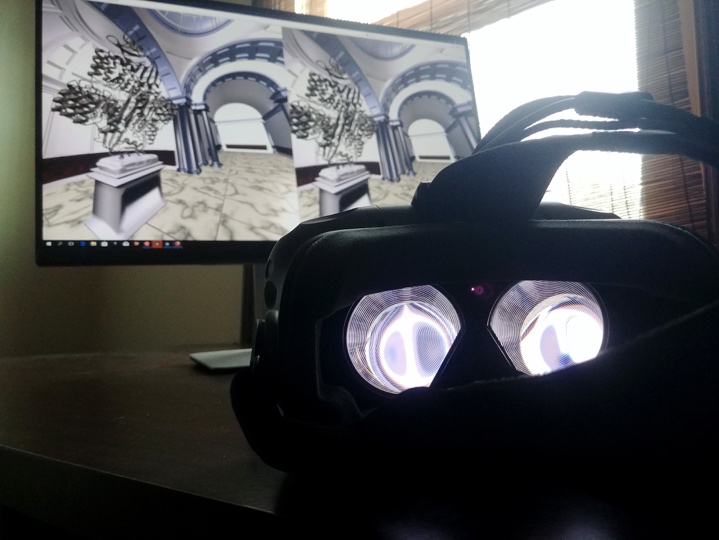


**Fig. S5. BlendMol enables virtual-reality macromolecular visualization in the browser.** We replaced Michelangelo's David with a model of the ribonuclease inhibitor-angiogenin complex.

After exporting the scene as a babylon file, we again wrote HTML/JavaScript code for browser-based visualization (Fig. S5, see Code S6 for the source code). Babylon.js includes easy functions for enabling WebVR. The user need only click the VR-headset icon in the lower-right corner of our web app’s screen. A guide sphere appears in the 3D scene when the user looks at the ground. To teleport to the sphere location, the user presses the spacebar or the VR-controller trigger. We successfully tested VR visualization in the Firefox browser, using an HTC Vive VR headset. Users without VR headsets can view and navigate the scene with a standard 2D monitor and keyboard. A live demo can be found at http://durrantlab.com/apps/blendmol/web/virtual-reality/

# Advanced Video Features

With BlendMol, users can also create macromolecular animations and videos. To illustrate, we created a video of the influenza neuraminidase enzymatic site. We first used the PDB2PQR server (Dolinsky, et al., 2007; Dolinsky, et al., 2004) to add hydrogen atoms to a crystal structure (PDB ID: 2HU4:A) (Russell, et al., 2006). After setting up the scene in VMD, we imported the VMD state file into Blender using BlendMol.

The Blender-rendered video demonstrates techniques that are not available in dedicated molecular-visualization programs (Video S2). For example, the radius of the simulated camera aperture is initially large, producing a strong Depth-of-Field effect (Fig. S6A). This effect diminishes as the camera approaches the bound small-molecule ligand (oseltamivir, an FDA-approved drug).

Several advanced lighting techniques were also used. To light the scene, we obtained and modified a public-domain, high-dynamic-range (HDR) image from hdrihaven.com. The video-game and film industries use HDR images (Akyüz, 2015) to capture the lighting details of real-world environments, often enhancing render realism. We also used bump mapping to create the textured protein surface evident in close-up shots (Fig. S6B).

Once the protein surface fades away, the viewer can see arginine residues that interact with the oseltamivir carboxylate group. To highlight one of these interactions, a sphere bounces between key atoms. The sparks left in its path were generated using Blender’s Particle System (Fig. S6C). Glowing cylinders then fade in to represent other arginine-oseltamivir interactions (Fig. S6D). Videos such as these could serve as powerful teaching tools.


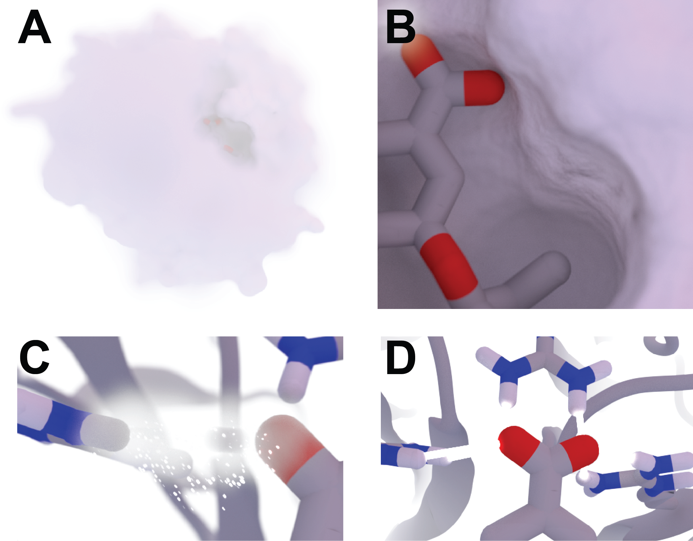


**Fig. S6.  Scenes taken from a molecular video.** A) Blender’s Depth-of-Field effect. B) Bump mapping imparts complex surface details that are not present in the actual mesh. C) Blender’s particle system. D) Glowing protein-ligand interactions.

# Pyrite-Based Molecular-Dynamics Visualization

BlendMol also simplifies use of our Pyrite Blender plugin (Rajendiran and Durrant, 2018). Pyrite coarse-grains a molecular-dynamics (MD) simulation over time (simulation frames) and space (atoms). It creates a Blender armature animation from the molecular motions of this simplified model. Parenting a previously imported macromolecular mesh to the armature imparts to it the same animation.

Pyrite requires the user to generate and import the mesh separately, and mesh and simulation coordinate systems must be identical. Unfortunately, VMD and PyMOL export meshes using coordinates that depend on the camera location/orientation. The user must run specific TCL or Python scripts prior to export to ensure that the coordinate systems match. BlendMol overcomes this limitation. If the user unclicks “Use nm, not Å?” in the import dialog, BlendMol imports the mesh with the proper, camera-independent coordinate system. Pyrite-generated animations can then be easily applied, without requiring separate VMD/PyMOL scripts.

To demonstrate, we used Pyrite and BlendMol to visualize a simulation of CDC73, a component of the RNA polymerase associated factor‐1 complex (Paf1C) (Amrich, et al., 2012). We used BlendMol to generate a mesh of the first-frame protein conformation within Blender. We then used Pyrite to animate that mesh per the MD simulation (357 frames). To further enhance the visualization, we imparted a marble material to the ribbon surface and used a mist pass to mimic fog. The resulting video is included in the supplementary material (Video S3).

# Comparing Other Modeling-Software Plugins

Blender itself ships with a simple PDB-importer plugin called Atomic Blender. Atomic Blender renders proteins as atom spheres connected by cylindrical bonds. In contrast, BlendMol has access to all the representations available in VMD and PyMOL, including surface and ribbon.

BioBlender (Andrei, et al., 2012; Zini, et al., 2010) is an another open-source Blender plugin for generating molecular meshes. In our initial tests, the plugin threw an error and only created many spheres centered at the origin. When we replaced the selenium atoms in our test PDB model (PDB ID: 1XDN) (Deng, et al., 2004) with sulfur atoms, the spheres were correctly positioned. We also used BioBlender to create a surface representation of the test protein. BlendMol has several notable advantages over BioBlender. First, our plugin generates molecular meshes by interfacing with well-tested visualization programs (i.e., VMD or PyMOL). It is thus less likely to suffer from case-specific bugs, such as the inability to deal with selenium atoms. Second, while BioBlender does include the surface representation, it lacks other popular representations. BlendMol provides more modelling options thanks to its VMD and PyMOL backends.

The Embedded Python Molecular Viewer (ePMV) (Johnson, et al., 2011) is an impressive open-source plugin for molecular visualization. In principle, it works in several 3D modeling programs, including Blender. ePMV provides a fuller range of molecular-visualization representations than many other plugins. Just as BlendMol interfaces with VMD and PyMOL, ePMV interfaces with the Python Molecule Viewer (Sanner, 2005). But VMD and PyMOL are more popular visualization programs. BlendMol is thus compatible with more common workflows. ePMV can also be difficult to install and does not appear to be compatible with the latest version of Blender.

Molecular Maya (mMaya, https://clarafi.com/tools/mmaya/) is another free plugin for advanced molecular visualization. It includes many of the same molecular representations that BlendMol provides (e.g., surface, stick, and ribbon representations). But mMaya works only in AutoDesk’s Maya modeling program, not Blender. Maya is a commercial product with a subscription model that costs over a thousand dollars a year.

# Limitations/Future Directions

BlendMol enables advanced macromolecular visualization. It is not an analysis tool and so is not meant to replace dedicated molecular-analysis programs such as VMD and PyMOL. This limitation is by design, as the Blender environment is poorly suited to atomic-resolution analyses.

When processing large or complex visualizations, VMD and PyMOL produce meshes that occupy large amounts of disk space. Importing these meshes into Blender is a time- and memory-consuming process. To speed import, we recommend using reduced-resolution representations when saving VMD state or PyMOL session files. Unchecking the “Remove Doubles” checkbox in the BlendMol dialogue is also helpful.

BlendMol is open source, and we encourage others to submit code updates addressing other limitations. Our code is well documented and should be easy to extend. We will also add new functionality as our ongoing visualization requirements change. For example, our plugin is incompatible with Chimera (Pettersen, et al., 2004), another popular stand-alone molecular-analysis program. We struggled to programmatically export meshes from Chimera using camera-independent coordinates. Future efforts will hopefully resolve this incompatibility. Several other advanced-visualization tasks, currently done manually, could also be automated. Journal images traditionally have white backgrounds, so most renders require a mist pass (i.e., fade to white). Camera focus is also useful for highlighting a specific molecular interaction or structure. Finding the ideal settings for these effects is tedious, as it depends on the distance and depth of the protein model relative to the camera. Automation of these processes will further simplify our macromolecular-visualization workflow.

Future efforts may also streamline the Blender-to-web workflow, allowing users to export browser-ready files directly from BlendMol. In the meantime, the HTML/JavaScript/CSS examples in the supplemental material (Code S5 and S6) should serve as useful guides.

References

Akyüz, A.O. High dynamic range imaging pipeline on the gpu. *Journal of Real-Time Image Processing* 2015;10(2):273-287.

Amrich, C.G.*, et al.* Cdc73 subunit of Paf1 complex contains C-terminal Ras-like domain that promotes association of Paf1 complex with chromatin. *J. Biol. Chem.* 2012;287(14):10863-10875.

Andrei, R.M.*, et al.* Intuitive representation of surface properties of biomolecules using BioBlender. *BMC Bioinf.* 2012;13 Suppl 4:S16.

Berman, H.M.*, et al.* The Protein Data Bank. *Nucleic Acids Res.* 2000;28(1):235-242.

Bommer, M.*, et al.* Crystallographic and Computational Analysis of the Barrel Part of the PsbO Protein of Photosystem II: Carboxylate-Water Clusters as Putative Proton Transfer Relays and Structural Switches. *Biochemistry* 2016;55(33):4626-4635.

Deng, J.*, et al.* High resolution crystal structure of a key editosome enzyme from Trypanosoma brucei: RNA editing ligase 1. *J. Mol. Biol.* 2004;343(3):601-613.

Dolinsky, T.J.*, et al.* PDB2PQR: expanding and upgrading automated preparation of biomolecular structures for molecular simulations. *Nucleic Acids Res.* 2007;35(Web Server issue):W522-W525.

Dolinsky, T.J.*, et al.* PDB2PQR: an automated pipeline for the setup of Poisson-Boltzmann electrostatics calculations. *Nucleic Acids Res.* 2004;32(Web Server issue):W665-W667.

Doppioslash, C. Making Shaders Artists Will Use. In, *Physically Based Shader Development for Unity 2017: Develop Custom Lighting Systems*. Berkeley, CA: Apress; 2018. p. 207-212.

Duwe, S.*, et al.* Expression-Enhanced Fluorescent Proteins Based on Enhanced Green Fluorescent Protein for Super-resolution Microscopy. *ACS Nano* 2015;9(10):9528-9541.

Johnson, G.T.*, et al.* ePMV Embeds Molecular Modeling into Professional Animation Software Environments. *Structure* 2011;19(3):293-303.

Lahr, R.M.*, et al.* The La-related protein 1-specific domain repurposes HEAT-like repeats to directly bind a 5'TOP sequence. *Nucleic Acids Res.* 2015;43(16):8077-8088.

McAuley, S.*, et al.* Practical physically-based shading in film and game production. In.: ACM; 2012. p. 10.

Papageorgiou, A.C., Shapiro, R. and Acharya, K.R. Molecular recognition of human angiogenin by placental ribonuclease inhibitor--an X-ray crystallographic study at 2.0 A resolution. *EMBO J.* 1997;16(17):5162-5177.

Pettersen, E.F.*, et al.* UCSF chimera - A visualization system for exploratory research and analysis. *J. Comput. Chem.* 2004;25(13):1605-1612.

Rajendiran, N. and Durrant, J.D. Pyrite: A blender plugin for visualizing molecular dynamics simulations using industry-standard rendering techniques. *J. Comput. Chem.* 2018;39(12):748-755.

Russell, R.J.*, et al.* The structure of H5N1 avian influenza neuraminidase suggests new opportunities for drug design. *Nature* 2006;443(7107):45-49.

Sanner, M.F. A component-based software environment for visualizing large macromolecular assemblies. *Structure* 2005;13(3):447-462.

Sanner, M.F., Olson, A.J. and Spehner, J.-C. Fast and robust computation of molecular surfaces. In.: ACM; 1995. p. 406-407.

Scalfani, V.F.*, et al.* Programmatic conversion of crystal structures into 3D printable files using Jmol. *Journal of cheminformatics* 2016;8(1):66.

Zini, M.F.*, et al.* Bioblender: Fast and efficient all atom morphing of proteins using blender game engine. *arXiv preprint arXiv:1009.4801* 2010.
