# Supplementary material for: BlendMol: advanced macromolecular visualization in Blender
Source: Bioinformatics. 2018 Nov 27;35(13):2323–5. doi: 10.1093/bioinformatics/bty968 (PMC6596883; doi:10.1093/bioinformatics/bty968)
Supplement: bty968_Supplementary_Data [file bty968_supplementary_data.zip › bty968-Suppl_data/CodeS5.Baked-Lighting-Shadows-In-Browser.zip!/CodeS2.Baked-Lighting-Shadows-In-Browser/index.html]

Light/Shadow Baking
